# Identification of Disalicyloyl Curcumin as a Potential DNA Polymerase Inhibitor for Marek’s Disease Herpesvirus: A Computational Study Using Virtual Screening and Molecular Dynamics Simulations

**DOI:** 10.3390/molecules28186576

**Published:** 2023-09-12

**Authors:** Aziza Cherif, Zarrin Basharat, Muhammad Yaseen, Mashooq Ahmad Bhat, Imad Uddin, Noha I. Ziedan, Fazal Mabood, Najla Sadfi-Zouaoui, Abdelmonaem Messaoudi

**Affiliations:** 1 Laboratoire de Mycologie, Pathologies et Biomarqueurs (LR16ES05), Département de Biologie, Université de Tunis-El Manar, Tunis 2092, Tunisia; cherifaziza96@gmail.com (A.C.); najla.sadfi@fst.utm.tn (N.S.-Z.); 2 Alpha Genomics Private Limited, Islamabad 45710, Pakistan; zarrin.iiui@gmail.com; 3 Institute of Chemical Sciences, University of Swat, Swat 19130, Pakistan; imadchemist1983@gmail.com (I.U.); fazal@uswat.edu.pk (F.M.); 4 Department of Pharmaceutical Chemistry, College of Pharmacy, King Saud University, Riyadh 11451, Saudi Arabia; mabhat@ksu.edu.sa; 5 Department of Physical Mathematical and Engineering Science, University of Chester, Chester CH2 4NU, UK; n.ziedan@chester.ac.uk; 6 Higher Institute of Biotechnology of Beja, Jendouba University, Habib Bourguiba Street, Beja 9000, Tunisia

**Keywords:** Marek’s disease, molecular docking, MDV DNA polymerase

## Abstract

Marek’s disease virus (MDV) is a highly contagious and persistent virus that causes T-lymphoma in chickens, posing a significant threat to the poultry industry despite the availability of vaccines. The emergence of new virulent strains has further intensified the challenge of designing effective antiviral drugs for MDV. In this study, our main objective was to identify novel antiviral phytochemicals through in silico analysis. We employed Alphafold to construct a three-dimensional (3D) structure of the MDV DNA polymerase, a crucial enzyme involved in viral replication. To ensure the accuracy of the structural model, we validated it using tools available at the SAVES server. Subsequently, a diverse dataset containing thousands of compounds, primarily derived from plant sources, was subjected to molecular docking with the MDV DNA polymerase model, utilizing AutoDock software V 4.2. Through comprehensive analysis of the docking results, we identified Disalicyloyl curcumin as a promising drug candidate that exhibited remarkable binding affinity, with a minimum energy of −12.66 Kcal/mol, specifically targeting the DNA polymerase enzyme. To further assess its potential, we performed molecular dynamics simulations, which confirmed the stability of Disalicyloyl curcumin within the MDV system. Experimental validation of its inhibitory activity in vitro can provide substantial support for its effectiveness. The outcomes of our study hold significant implications for the poultry industry, as the discovery of efficient antiviral phytochemicals against MDV could substantially mitigate the economic losses associated with this devastating disease.

## 1. Introduction

Marek’s disease virus (MDV), caused by Gallid alphaherpesvirus 2 (GaHV-2), was described for the first time in 1907 by Joseph Marek. It is a highly contagious alphaherpesvirus that induces neurological lesions, immune suppression, and tumor proliferation of lymphoid cells in poultry [1]. MDV infects chickens and causes cancer, with significant economic consequences for the poultry industry. Annual economic losses worldwide totaled USD 2 billion [2]. MDV is a cell-associated and double-stranded DNA virus that belongs to the family of Herpesviridae, the subfamily of Alphaherpesvirinae, and the genus Mardivirus. The latter comprises four other species: the Gallid herpesvirus 3 (GaHV-3), the Meleagrid herpesvirus 1 (MeHV-1), the Anatid herpesvirus 1 and the Columbid herpesvirus 1 [3]. A recent survey indicates that MDV remains a matter of serious concern in many countries due to the emergence of very virulent pathotypes [1]. Vaccination is the main preventive measure against MDV for the poultry industry. The use of MDV attenuated viral strains in the 1970s was followed by the HVT/MDV-2 bivalent vaccine in the 1980s to protect against emerging MDV pathotypes [4]. Despite widespread vaccination, Marek’s disease virus has shown a continuous increase in its virulence and has acquired the ability to overcome immune responses induced by such vaccines [5].

The effectiveness of various vaccine types is impacted by the evolution of the related infectious pathogens. Specifically, replication, a crucial and essential biological process that provides an important basis for the development of new antiviral drugs, is still very much an ongoing process, and it is considered a leading solution to the vaccine resistance problem [6]. These molecules must target key stages of the viral therapeutic target. Therefore, viral polymerases, key enzymes that play a central role in viral genome replication and transcription, are an extremely favorable target for antiviral therapy [7].

DNA polymerase is required for viral replication, and this enzyme with a relatively conserved sequence and structural features is considered an extremely favorable target for the development of antiviral drugs against numerous diseases, including animal viral infection [8]. Many studies report the use of acyclovir, a synthetic nucleoside analogue with a higher affinity for viral DNA polymerase, which is widely used in the treatment of herpesvirus infections, particularly the herpes simplex virus (HSV) and varicella-zoster virus (VZV) [9]. Many nucleoside analogs are also known for their ability to target activity of prokaryotic and eukaryotic DNA polymerases such as AZT (3-azidothymidine) [10] or fludaribine [11].

Phytomolecules from medicinal and aromatic plants with promising anticancer and antiviral applications represent a huge arsenal that can be used against many animal or fowl viral diseases [12]. The success of herbal medicinal therapy is due to its lower or less severe toxicity and side effects [13]. Numerous studies documented the antiviral potential of several plant extracts against viral pathogens of domestic birds, particularly poultry. The medicinal plants *O. compressa*, *N. procumbens*, and *S. surattense* were found to be more effective to treat avian influenza disease [14]. The efficacy of the Aloe species, *Azadirachta indica*, and *Commiphora swynnertonii* plant extract has been experimentally confirmed in the management and treatment of the Newcastle disease virus (NDV) [15].

Recently, computer aided drug design approaches became more frequently used as they screen a higher number of compounds in shorter durations and at lower costs using different methods. Structure-based drug design is the process used to design and optimize a series of potent inhibitors from a large chemical library using structural information of a biological target [16]. Due to the ongoing evolution of virulence in MDV strains, leading to immune failure, a successful treatment for the highly virulent pathotype remains elusive [17]. In the absence of a specific therapy, there is an urgent need to discover new, effective, and safe treatments to monitor this outbreak. Here, we used a variety of computational methods to target the MDV DNA polymerase enzyme to identify a new potential plant-derived antiviral agent to improve MDV vaccine efficacy for better controlling the MDV viral infection.

## 2. Results

### 2.1. Prediction of the 3D Structure of MDV DNA Polymerase

**Figure 1:**
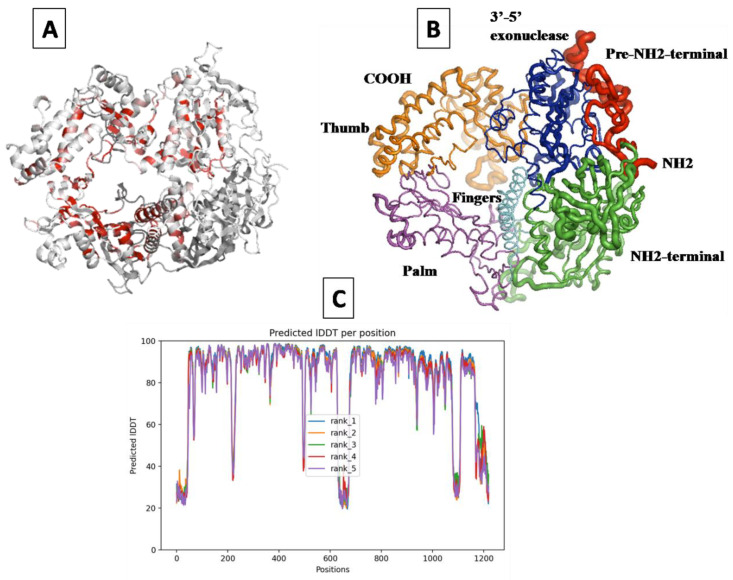
The predicted structure of the MDV DNA polymerase enzyme. (**A**) The cartoon representation of the MDV DNA polymerase colored as a function of similarity scores. This color ramping from white (low score) to red (identity) allows to quickly locate areas of weak and strong sequence conservation. (**B**) Overall structure of the predicted model of the MDV DNA polymerase in ribbon diagram. Pre-NH2 Domain (41–116), NH2-terminal domain (141–362 and 603–665), 3′–5′-exonuclease domain (347–602), palm subdomain (666–749 and 811–924), finger subdomain (750–810), and thumb subdomain (925–1170) are colored red, green, blue, magenta, cyan, and orange, respectively. (**C**) The predicted local-distance difference test (pLDDT) value over 1120 residues of the MDV DNA polymerase enzyme (the higher the value is, the better confidence the model has).

Using AlphaFold, the 3D structure of MDV DNA polymerase was accurately predicted with high precision, as evidenced by the majority of residues receiving a pLDDT score of 95 (Figure 1C). Furthermore, 85.8% of the residues were situated in the most favorable region of the Ramachandran plot, while 11.9%, 1.7%, and 0.6% were in the allowed, generously allowed, and disallowed regions, respectively, indicating that the predicted model had good stereochemical quality.The overall quality factor predicted by ERRAT was 92.41%, which is generally accepted to indicate a high-quality model when the value is greater than 50. Additionally, the analysis by Verify3D showed that 72.04% of the target residues had an average score of 3D − 1D > 0.2, confirming the compatibility of the 3D structure with its amino acid sequence (Figure S1, Supplementary Material).

The multiple sequence alignmenet of the 20 most homologous proteins to the MDV DNA polymerase model, obtained through a BLAST+ search against the PDB database, revealed the top-listed homologousprotein structures, including 2GV9 and 7LUF, which represent the human crystal structure of the human Herpes Simplex virus type 1 DNA polymerase. The cartoon depiction of the modeled structure is colored based on similarity scores determined from the previous MSA, with the color ramping from white (low score) to red (identity), allowing for easy identification of areas of weak and strong sequence conservation on the modeled structure (Figure 1A).

Since the two enzymes share a 53% sequence similarity, the 3D structures of Marek’s disease virus DNA polymerase and human Herpes Simplex Virus 1 DNA polymerase are very similar. The MDV DNA polymerase revealed a conserved B-family architecture composed of Six structural domains: the pre-NH2 domain, the NH2-terminal domain, the 3′–5′ exonuclease domain, the palm subdomain, the finger subdomain, and the thumb subdomain. These domains form a ribbon-like structure with a hole in the middle, which encircles the primer-template DNA. The NH2 and COOH termini are located on opposite sides of the protein (Figure 1B).

### 2.2. Virtual Screening and Docking

The Autodock Tools software was used to prepare the target by adding polar hydrogen bonds and Kollman charges, followed by subsequent energy minimization. The ligand library was composed of several thousand anti-viral drugs known for their efficacy against HSV, as well as phytochemicals and natural-like compounds. Based on the docked poses within the predicted active sites of the MDV DNA polymerase, the top molecules with a lower docking score were identified. The docked conformation with the lowest docking score for each compound was chosen, and their interactions with the functional active site residues were investigated. The top 30 ligands ranked by docking score are listed in Table 1.

Based on the docking results, the top 5 compounds that had a good binding affinity with the target included the curcumin analogs Disalicyloyl curcumin, Ferrocenyl curcumin, Curcumin Dimer 1, Curcumin Dimer 2, and 4-(4-Hydroxybenzylidene) curcumin. The corresponding binding energies are −12.66 kcal/mol, −11.04 kcal/mol, −10.54 kcal/mol, −10.26 kcal/mol, and −10.09 kcal/mol. Table 2 shows the estimated binding energy, structure 2D, amino acids involved in the interaction, and a schematic of detailed ligand atom interactions of the top five ligands with the protein residues.

When docked with the MDV DNA polymerase receptor, disalicyloyl curcumin showed a single hydrogen bond with acidic Glu521 residue and a non-covalent cation–π interaction (with basic residue Lys501) (Table 2). O22 atom of the ferrocenyl curcumin displayed hydrogen interactions with Lys501 (3.78 angstrom, −1.1 kcal/mol) and Lys502 (3.18 angstrom, −6.2 kcal/mol). Ionic interactions were observed between C5 and C21 atom of the ligand and Lys501 of the protein (with distances 3.88 and 3.99 angstrom; energy values −0.5 and −0.7 kcal/mol, respectively). Curcumin dimer 1 showed several hydrogen bonds and an ionic interaction with Lys 501. Gln 500 acted as a hydrogen acceptor and donor with atom O31 of the curcumin dimer 2, with a distance of 3.01 (energy value: −0.7 kcal/mol) and 3.04 angstrom (energy value: 3.04 kcal/mol), respectively.

Given that disalicyloyl curcumin has the best binding energy score (−12.66 Kcal/mol), it is suggested that it could be an inhibitor that is more effective and selective towards the target. This energy is the result of the formation of a hydrogen bond between the residue Glu521, and ligand, which is spaced apart by 3.04 angstroms, with an energy value of −0.8 kcal/mol. Four residues engaged in other interactions to further stabilize the complex.

### 2.3. Molecular Dynamics Simulation

**Figure 2:**
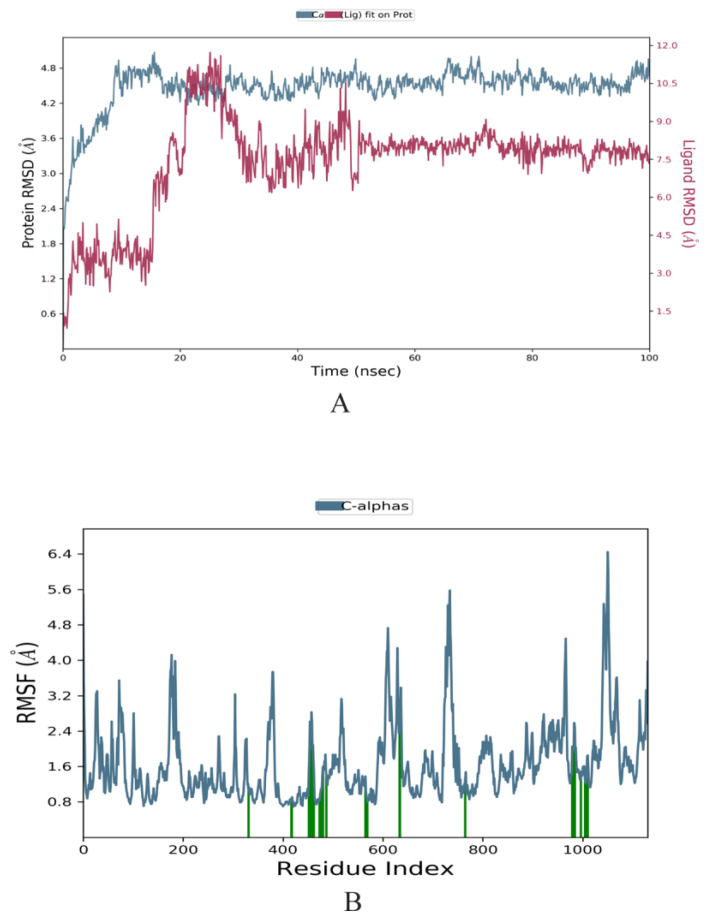
(**A**) Analysis of residual flexibility via root-mean-square deviation (RMSDs) over a time period of 100 ns. (**B**) Analysis of residual flexibility via root-mean-square fluctuations (RMSFs) over a time period of 100 ns.

Based on docking results, an MD simulation was carried out using Desmond to examine the stability of the MDV DNA polymerase-Disalicyloyl curcumin complex. The RMSD (Root mean square deviation) calculated during 100 ns simulations for the top complex revealed a trajectory with average RMSD between 3 Å and 4.8 Å (Figure 2A). Although there were little deviations up to 20 ns, the complex stabilized afterwards. Ligand RMSD was stable around 7.5 Å.

The root-mean-square fluctuation (RMSF) is useful for characterizing local changes along the protein chain. Figure 2B shows peaks that indicate areas of the protein that fluctuate the most during the simulation. The MDV DNA polymerase had an average RMSF of 1.6 for overall positions. Overall, it can be inferred that MDV DNA polymerase forms a favorable interaction with the disalicyloyl curcumin ligand, resulting in the creation of a highly stable complex.

**Figure 3:**
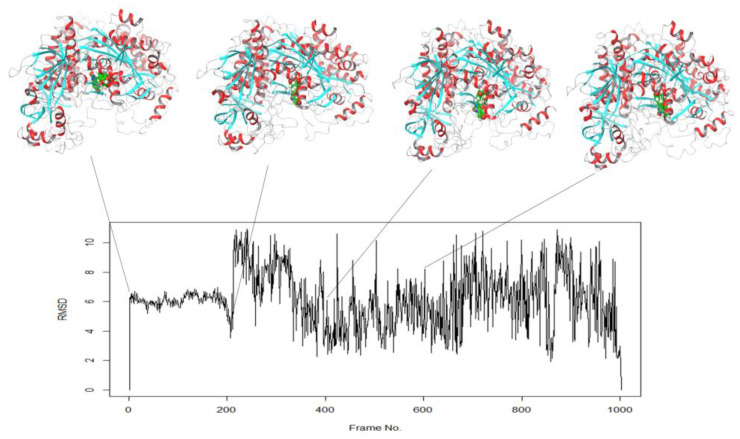
Altered position of ligand in the complex correlates with RMSD of the various frames of simulation trajectory. The protein is shown in ribbon (red = helices, sky blue = beta-sheets), while the ligand is shown in CPK space filled form.

To gain further insights into the observed fluctuations in RMSD during the MD simulation, we examined the correlation between the RMSD values and the position of the ligand. We found that the complex underwent a large conformational change at around 20 ns of simulation time, which resulted in a significant increase in RMSD. The position of the ligand was also found to be altered at frames 200, 400, and 600, corresponding to 20, 40, and 60 ns of simulation time, as visualized in Figure 3.

**Figure 4:**
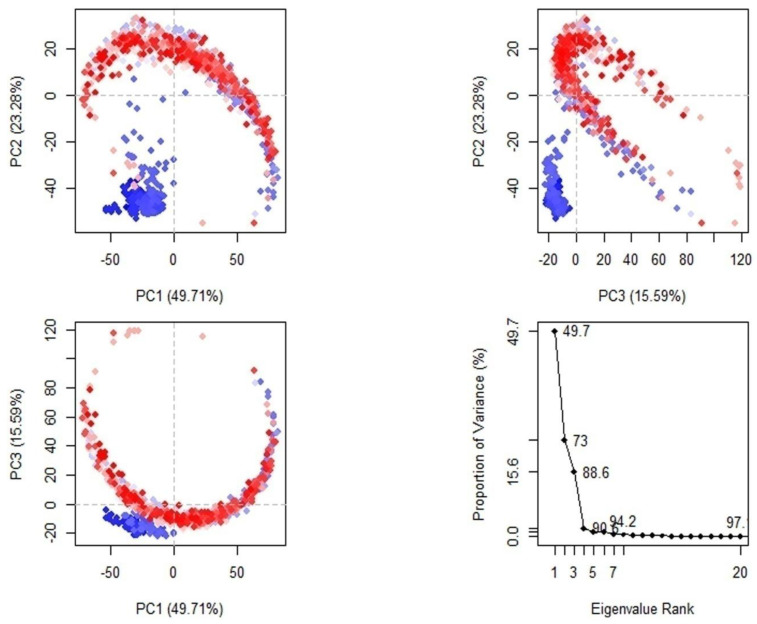
PCA plots of the simulated MDV DNA polymerase. The trajectory frames are colour coded, shown by transition of blue to white to red with passage of time. Blue shows low, while red shows high atomic displacements.

To analyze the conformational changes more rigorously, we performed a principal component analysis (PCA) and plotted the results in a three-dimensional space (Figure 4). The PCA plots clearly show variations in the positioning of the MDV DNA polymerase along PC1, PC2, and PC3, indicating multiple conformational changes occurring throughout the simulation. These findings suggest that the interactions between the MDV DNA polymerase and Disalicyloyl curcumin are dynamic and complex, leading to significant changes in the overall structure of the complex over the course of the simulation.

### 2.4. ADMET Prediction of Compounds

Disalicyloyl curcumin stood out as having relatively better Caco2 permeability and unbound fraction in the blood, free to interact with tissues (Table 3), making it an interesting compound for drug development. Intestinal absorption was best for ferrocenyl curcumin, followed by N-(4 Methoxyphenylpyrazole) and disalicyloyl curcumin. Ferrocenyl curcumin and N-(4 Methoxyphenylpyrazole) curcumin were hepatotoxic, while the rest were non-toxic. In light of several parameters, disalicyloyl curcumin appeared as a good drug candidate.

Using the ProTox-II online program, we performed additional toxicity prediction studies to look at the safety profile of the disalicyloyl curcumin ligand. This server divided substances into six categories based on their levels of toxicity, with class 1 being the most lethal and toxic with an estimated lethal dosage (LD50) of 5, and class 6 showing an LD50 > 5000, indicating the compound is non-toxic.Disalicyloyl curcumin was classified as belonging to class 4 by ProTox-II with an LD50 of 500 mg/kg; this analysis also shows that the drug is not hepatotoxic and does not represent a significant risk of mutagenicity, carcinogenicity or cytotoxicity. However, since the disalicyloyl curcumin compound overcame all toxicity barriers, it can be considered a potential bioactive molecule.

## 3. Discussion

Marek’s disease (MD) is a severe and fatal lymphoproliferative disease of chickens that causes hugeeconomic loss in the poultry industry. The current vaccine for MDV does not provide sterile immunity, which may lead to the emergence of more resistant and virulent strains of the virus. This highlights the urgent need to develop more effective therapeutics that inhibit virus replication and improve the health and welfare of these birds to secure the global food supply [18]. Plants offer an abundant source of novel compounds with the potential to treat a wide range of diseases. The vast collection of medicinal plants available today includes those with a broad spectrum of antiviral activity against chicken viruses like influenza virus and Newcastle disease virus. Despite this, there has been a notable lack of research investigating the potential of medicinal plant compounds against Marek’s Disease Herpesvirus [19] Therefore, a computational study using virtual screening and molecular dynamics simulations can provide an opportunity to explore this untapped resource and identify promising new compounds for the treatment of this devastating poultry disease.

In this study, the accurate prediction of the three-dimensional structure of MDV DNA polymerase using AlphaFold has enabled us to gain insights into the structural and functional features of this important enzyme. Virtual screening and docking experiments were conducted to identify potential inhibitors of MDV DNA polymerase.

The virtual screening and docking experiments conducted in the study identified curcumin analogs as potential inhibitors of MDV DNA polymerase, which could lead to the development of new antiviral drugs for the treatment of MDV infections. The identified curcumin analogs may be effective inhibitors of MDV DNA polymerase by binding to the active site of the enzyme and inhibiting its activity. This could potentially halt the replication of the virus, leading to reduced viral load and alleviation of symptoms.

Curcumin analogs have been shown to have potent antiviral activity against a variety of avian viruses and have been investigated for their potential use in drug development. In addition, curcumin has been studied for its potential use in reducing enteric isoprostane and prostaglandin levels in chickens infected with Eimeria maxima. A study conducted by Shehata et al. found that chickens infected with Eimeria maxima and administered with curcumin had reduced enteric isoprostane 8-iso-PGF2α and prostaglandin GF2α levels. The study also showed a reduction in Salmonella enterica serovar Typhimurium intestinal colonization and improved intestinal homeostasis in the treated chickens [20].

One of the major challenges in using curcumin as a therapeutic agent is its poor bioavailability, which limits its clinical efficacy. However, a potential solution to this problem is the use of nanocapsules to improve the delivery and absorption of curcumin. Nanocapsules are small particles that can encapsulate the active ingredient and protect it from degradation or metabolism. This can increase the stability and solubility of curcumin, allowing for better absorption and delivery to target tissues. Several studies have explored the use of nanocapsules as a strategy to improve the bioavailability of curcumin. These studies have shown promising results, with increased absorption and efficacy observed in animal models [21].

Overall, the study presented here highlights the enormous potential of computational methods in providing valuable insights into the structure and function of the MDV DNA polymerase, an essential therapeutic target for the development of new treatments against Marek’s Disease Herpesvirus (MDV) in chickens. By identifying curcumin analogs as promising inhibitors of this critical viral enzyme, this study has opened up new possibilities for the development of effective therapies against MDV in chickens.

## 4. Materials and Methods

### 4.1. Three-Dimensional Structural Prediction

The amino acid sequence of the MDV DNA polymerase was retrieved from the UniPort server (ID: Q9E6N9) in June 2022. This sequence of 1120 amino acids was obtained from the first complete genomic sequence of a highly virulent strain of Marek’s disease virus serotype 1 (MDV1), Md5 [22]. The three-dimensional structure of the MDV DNA polymerase was predicted using AlphaFold, an AI system developed by DeepMind that predicts a protein’s 3D structure with high quality from its amino acid sequence [23]. The protein sequence (UniProt ID: Q9E6N9) was uploaded to the Google Colab version of AlphaFold in June 2022, and the resulting structure was analyzed using a variety of validation methods.

To assess the quality of the model, AlphaFold produces a per-residue estimate of its confidence on a scale from 0 to 100, known as the pLDDT score. Regions with pLDDT > 90 are expected to be modeled with high accuracy, while those with pLDDT between 50 and 70 have low confidence and should be treated with caution. In our study, we used pLDDT scores to evaluate the quality of the model.

We also used the Structural Analysis and Verification Server (SAVES) to evaluate the quality of the model [24]. SAVES is a metaserver that runs six different programs for validating the submitted protein structure. Specifically, we used the Ramachandran plot to check the stereochemical quality of the protein structure [25], ERRAT to evaluate the statistics of non-bonded interactions between different atom types [26], and Verify 3D to analyze the compatibility of the atomic 3D model with its own amino acid sequence [27]. Together, these methods provided a comprehensive assessment of the quality of the predicted protein structure.

### 4.2. Target Preparation and Active Site Analysis

To predict the active site of our modeled polymerase, we used the Computed Atlas of Surface Topography of Proteins (CASTp) tool [28]. CASTp provides detailed information about the surface and volume of pockets, interior cavities, and cross channels, as well as an estimation of the binding site’s accessibility to other molecules and a detailed specification for the amino acids responsible for active site formation inside our proteins [29].

For target preparation, we used the Autodock Tools software [30]. First, we added polar hydrogen bonds and Kollman charges to initialize the target for the docking process. Then, we performed energy minimization to remove bad steric clashes. The final obtained file was saved in the pdbqt format.

### 4.3. Ligand Preparation

A ligand library of potential MDV DNA polymerase inhibitors was built after a literature survey. Various studies are examined, and the final ligand library was composed of several thousand anti-viral drugs known for their remarkably powerful efficacy against HSV. The list includes derivatives of Acyclovir, Ivermectin, Foscarnet, Favipiravir, Benzothiazoles, and Withaferin. The 2D structures of these molecules were obtained from the PubChem database (https://pubchem.ncbi.nlm.nih.gov/; accessed on 6 March 2023) and from ZINC database (https://zinc.docking.org/; accessed on 8 March 2023) (n = 16,287). Phytochemicals and Natural-Like Compounds were also screened against MDV DNA polymerase, with compounds retrieved from the African Natural Products Database (http://african-compounds.org/nanpdb/; accessed on 12 March 2023) (n = 6515), the Natural Products Occurrence Database LOTUS (https://lotus.naturalproducts.net/; accessed on 12 March 2023) (n = 276,518), andthe traditional Chinese medicine database (n = 36,043) compounds from ZINC database.

### 4.4. In Silico Screening of the Potential MDV DNA Polymerase Inhibitors

Following the preparation phase, the selected ligands were docked into the modeled polymerase’s catalytic site. We set a grid box with dimensions of 60, 60, 60 to cover the entire enzyme binding site, and it was centered at x, y, and z = 19.015, 45.391, and 14.367, respectively. Molecular docking was carried out using the Lamarckian genetic algorithm (LGA) in AutoDock 4.2. We used an initial population size of 150 individuals, the maximum number of generations set at 27,000, and a gene mutation rate of 0.02 with a probability that follows a Cauchy law (mean 0, variance 1). After completing the screening of the ligand library by estimating their conformation and orientation at the MDV DNA polymerase active site using a molecular docking iterative process, we selected the conformation with the lowest docked energy. We further analyzed the binding interactions of the best target-ligand complex conformations, including hydrogen bonds and bond lengths, usingMOE software v2016. ADMET values were predicted using pKCSM [31].

### 4.5. Molecular Dynamics Simulation

To explore the conformational space of the protein–ligand complex, molecular dynamics (MD) simulation was performed using the classical mechanics approach. The docked complex was prepared in Tip3P water solvated box for MD simulation in the Schrodinger Desmond module (Desmond Molecular Dynamics System, D. E. Shaw Research, New York, NY, USA, 2021). The system was evolved over time for 100 ns, and the stability of the protein–ligand complex was investigated. The movement of the protein docked to the predicted top inhibitory compound was examined and analyzed [32].

### 4.6. Post-MD Analysis

Post-MD analysis was performed using the Bio3D module of the R package [33]. The trajectory was converted to .dcd format and aligned to reference frame 0 using the fit.xyz() function. RMSD and principal component analysis (PCA) were plotted. PCA is a widely used technique for analyzing the conformational dynamics of biomolecules, including protein–ligand complexes. The covariance matrix was calculated by the cov.xyz() function and its principal components by the pca.xyz() function.

### 4.7. Toxicity Predictions

The toxicological parameters of the on top-scored compounds were predicted using The Protox II webserver (https://tox-new.charite.de/protox_II/; accessed on 21 March 2023) [34]. The Protox II webserver uses a machine learning-based approach to predict toxicity endpoints for small molecules based on their chemical structure. The toxicity endpoints predicted include hepatotoxicity, mutagenicity, immunotoxicity, carcinogenicity, and acute oral toxicity. To perform the toxicity predictions, the SMILES strings for the top-scored compounds were submitted to The Protox II webserver, and the predicted toxicity endpoints were obtained. The details of the method used by The Protox II webserver are available elsewhere [34].

## 5. Conclusions

We have identified a novel potential DNA polymerase inhibitor for Marek’s disease herpesvirus by applying a computer-aided drug design protocol involving prediction based on AlphaFold system and structure-based virtual screening with docking simulations. This study indicates the involvement of hydrophobic interactions between a DNA polymerase and disalicyloyl curcumin, a derivative of curcumin with a binding energy of −12.66 kcal/mol. These findings suggest that disalicyloyl curcumin can act as an effective and potential inhibitor for Marek’s disease DNA polymerase. Further in vitro and in vivo validation will help us to understand the molecular mechanism of this curcumin derivative on this target.

## Figures and Tables

**Table 1:** Top 30 ligands ranked by docking score.

S No	Compound	Binding Energy (Kcal/mol)
1	Disalicyloyl curcumin	−12.66
2	Ferrocenyl curcumin	−11.04
3	Curcumin dimer 1	−10.54
4	Curcumin dimer 2	−10.26
5	N-(4 Methoxyphenylpyrazole)Curcumin	−10.09
6	4-(4-Hydroxybenzylidene) curcumin	−9.91
7	Phenyl Diphenoxyphosphinecarboxylate Oxide	−9.66
8	Curcumin dimer 3	−8.94
9	Turmeric	−8.90
10	Curcumine-difluorée	−8.12
11	4-Benzylidene Curcumin	−8.11
12	Di-(tert-Butyl-dimethylsilyl) Curcumin	−8.01
13	Withaferin A	−7.74
14	1-Benzothiophene–2-Sulfonamide	−6.60
15	Phosphinecarboxylic acid, dihydroxy-, 4-chlorophenyl ester, oxide	−6.53
16	Ribasphere	−6.48
17	Sodium Phenyl Phenoxycarbonylphosphonate	−6.29
18	Methyl [(4-acetylphenoxy)-methoxyphosphoryl]	−6.28
19	Methyl [(4-acetylphenoxy)-methoxyphosphoryl]	−6.22
20	Adenosine	−6.18
21	Tert-butyl 6-fluoro–1H-pyrazolo[3,4-b] pyridine−3-carboxylate	−5.51
22	(4-chlorophenoxy)carbonyl ethoxyphosphinic acid	−4.30
23	Elvucitabine	−4.21
24	4-[(E)-(4-Chlorophenyl)methylideneamino]–3-(2,4-dichloro–5-fluorophenyl)–1H–1,2,4-triazole–5-thione	−4.08
25	6-Amino–3-[(E)-[2-fluoro–2 (hydroxyméthyl)cyclopropylidène]méthyl]–1,6-dihydropyrimidine–2-one	−3.80
26	8-Oxo-Dgtp	−3.65
27	Acyclovir Triphosphate	−3.22
28	DisodiumMethox phosphinecarboxylate Oxide	−3.15
29	Isopropyl Dimethoxyphosphinecarboxylate Oxide	−3.07
30	CyclopentylmethylDimethoxyphosphinecarboxylate Oxide	−3.04

**Table 2:** Estimated Binding Energy, 2D Structure, amino acids involved in interaction and diagrammatic sketch of top five ligands. (3D depiction shown in Figure S2, Supplementary Material.)

Compound	Structure 2D	Binding Affinity (kcal/mol)	H Bond Residues	Other Interactions	Diagrammatic Sketch Illustrating the Interactions
Disalicyloyl curcumin	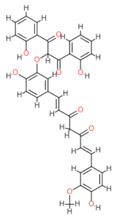	−12.66	Lys 501 Glu 521	Gln 500 Lys 502 Ser 517 Tyr 1037	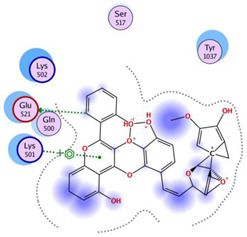
Ferrocenyl curcumin	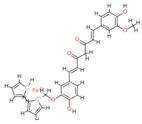	−11.04	Lys501 Lys 502	Gln 500 Ser 517 Ile 518 Thr 520 Glu 521 Ile 603	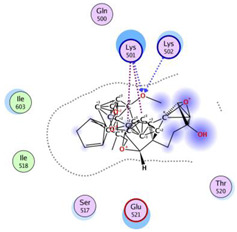
Curcumin dimer 1	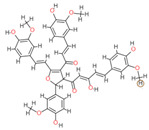	−10.54	Gln 500 Lys 501 Lys 502 Thr 520 Ser 517	Phe 499 Ile 518 Gln 521 Gln 607	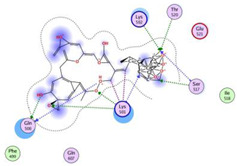
Curcumin dimer 2	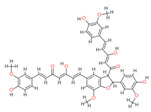	−10.26	Gln 500	Phe 499 Lys 501 Lys 502 Gly 503 Ser 517 Ile 518 Glu 521 Ile 603 Phe 604 Gln 607 Tyr 799 Tyr 1037	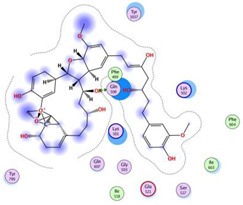
N-(4 Methoxyphenylpyrazole) Curcumin	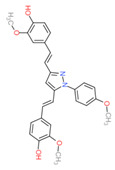	−10.09	Lys 502 Ser 517	Gln 500 Lys 501 Ile 518 Thr 520 Glu 521 Tyr 1037	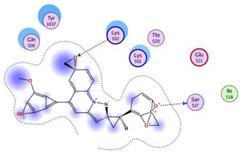

**Table 3:** ADMET profile of prioritized compounds.

Property	Model Name	Disalicyloyl Curcumin	Ferrocenyl Curcumin	Curcumin Dimer 1	Curcumin Dimer 2	N-(4 Methoxyphenylpyrazole) Curcumin	Unit
Absorption	Water solubility	−3.182	−4.172	−2.984	−2.967	−4.139	Numeric (log mol/L)
	Caco2 permeability	0.019	−0.248	−0.699	−0.786	0.604	Numeric (log Papp in 10^−6^ cm/s)
	Intestinal absorption (human)	81.841	95.281	75.414	76.116	91.876	Numeric (% Absorbed)
	Skin Permeability	−2.735	−2.735	−2.735	−2.735	−2.735	Numeric (log Kp)
	P-glycoprotein substrate	Yes	Yes	Yes	Yes	Yes	Categorical (Yes/No)
	P-glycoprotein I inhibitor	Yes	Yes	Yes	Yes	Yes	Categorical (Yes/No)
	P-glycoprotein II inhibitor	Yes	Yes	Yes	Yes	Yes	Categorical (Yes/No)
Distribution	VDss (human)	−1.632	−1.123	−1.685	−1.648	−1.183	Numeric (log L/kg)
	Fraction unbound (human)	0.168	0.01	0.149	0.141	0.052	Numeric (Fu)
	BBB permeability	−1.532	−0.668	−1.929	−2.042	−0.765	Numeric (log BB)
	CNS permeability	−3.504	−2.685	−3.123	−3.079	−2.647	Numeric (log PS)
Metabolism	CYP2D6 substrate	No	No	No	No	No	Categorical (Yes/No)
	CYP3A4 substrate	Yes	Yes	Yes	Yes	Yes	Categorical (Yes/No)
	CYP1A2 inhibitior	No	No	No	No	No	Categorical (Yes/No)
	CYP2C19 inhibitior	No	Yes	No	No	Yes	Categorical (Yes/No)
	CYP2C9 inhibitior	Yes	Yes	No	No	Yes	Categorical (Yes/No)
	CYP2D6 inhibitior	No	No	No	No	No	Categorical (Yes/No)
	CYP3A4 inhibitior	Yes	Yes	Yes	Yes	Yes	Categorical (Yes/No)
Excretion	Total Clearance	−0.153	0.864	−0.132	−0.038	0.09	Numeric (log ml/min/kg)
	Renal OCT2 substrate	No	No	No	No	No	Categorical (Yes/No)
Toxicity	AMES toxicity	No	No	No	No	No	Categorical (Yes/No)
	Max. tolerated dose (human)	0.37	−0.034	0.391	0.377	0.087	Numeric (log mg/kg/day)
	hERG I inhibitor	No	No	No	No	No	Categorical (Yes/No)
	hERG II inhibitor	Yes	Yes	Yes	Yes	Yes	Categorical (Yes/No)
	Oral Rat Acute Toxicity (LD50)	2.638	2.016	2.529	2.53	2.522	Numeric (mol/kg)
	Oral Rat Chronic Toxicity (LOAEL)	2.508	2.08	3.069	3.331	2.226	Numeric (log mg/kg_bw/day)
	Hepatotoxicity	No	Yes	No	No	Yes	Categorical (Yes/No)
	Skin Sensitisation	No	No	No	No	No	Categorical (Yes/No)
	*T. pyriformis* toxicity	0.285	0.286	0.285	0.285	0.286	Numeric (log ug/L)
	Minnow toxicity	−0.668	−3.33	−2.905	−3.603	−2.349	Numeric (log mM)

## Data Availability

Not applicable.

## Supplementary Materials

The following supporting information can be downloaded at: https://www.mdpi.com/article/10.3390/molecules28186576/s1, Figure S1: Validation of the 3D model of the MDV DNA polymerase enzyme (A) Distribution of amino acid residues according to their average scores to assess the structural quality of the predicted homology model. (B) Ramachandran plot. (C) ERRAT analysis of the predicted 3D model of the MDV DNA polymerase the black bars identify the misfolded region, the gray bars correspond to the error region between 95% and 99% and the white bars indicate the region with a lower error rate for protein folding; Figure S2: 3D complex of the drug target with (A) Disalicyloyl curcumin, (B) Ferrocenyl curcumin (C) Curcumin dimer 1 (D) Curcumin dimer 2 (E) N-(4 Methoxyphenylpyrazole) Curcumin.
