# Supplementary material for: Identification of Disalicyloyl Curcumin as a Potential DNA Polymerase Inhibitor for Marek’s Disease Herpesvirus: A Computational Study Using Virtual Screening and Molecular Dynamics Simulations
Source: Molecules. 2023 Sep 12;28(18):6576. doi: 10.3390/molecules28186576 (PMC10537106; doi:10.3390/molecules28186576)
Supplement: Supplementary file 1 [file molecules-28-06576-s001.zip › molecules-2489076-supplementary.pdf]

(A)

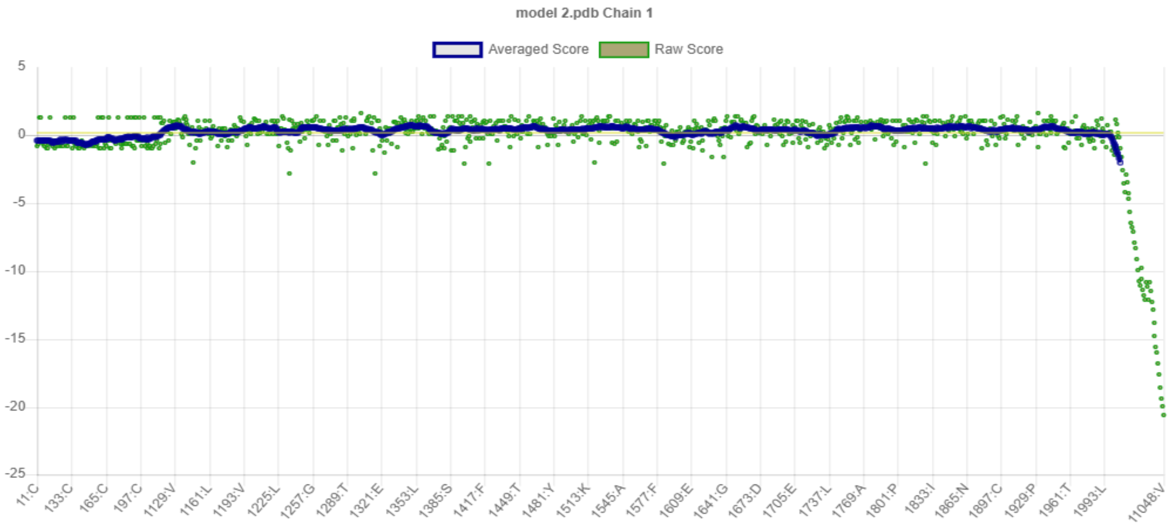

(B)

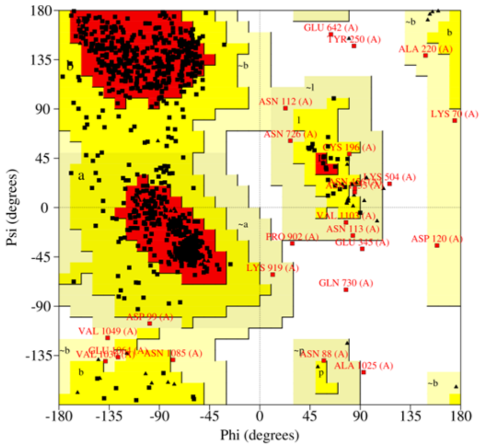

Plot statistics

Residues in most favoured regions [A,B,L]	870	85.8%
Residues in additional allowed regions [a,b,l,p]	121	11.9%
Residues in generously allowed regions [~a,~b,~l,~p]	17	1.7%
Residues in disallowed regions	6	0.6%
	----	------
Number of non-glycine and non-proline residues	1014	100.0%
Number of end-residues (excl. Gly and Pro)	2	
Number of glycine residues (shown as triangles)	60	
Number of proline residues	48	

Total number of residues	1124	

Based on an analysis of 118 structures of resolution of at least 2.0 Angstroms and R-factor no greater than 20%, a good quality model would be expected to have over 90% in the most favoured regions.

(C)

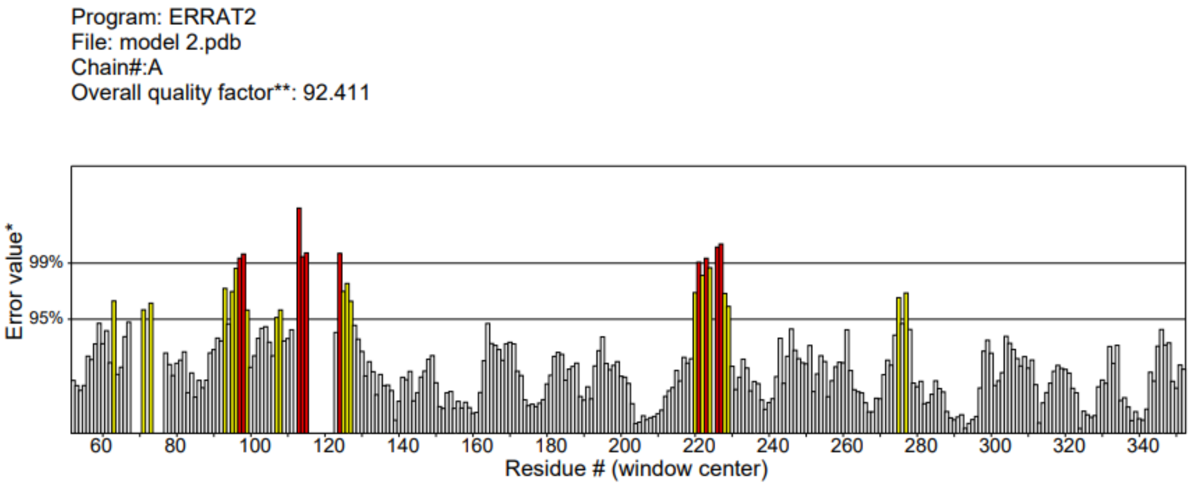

Figure S1: Validation of the 3D model of the MDV DNA polymerase enzyme **(A)** Distribution of amino acid residues according to their average scores to assess the structural quality of the predicted homology model**. (B)** Ramachandran plot. **(C)** ERRAT analysis of the predicted 3D model of the MDV DNA polymerase The black bars identify the misfolded region, the gray bars correspond to the error region between 95% and 99% and the white bars indicate the region with a lower error rate for protein folding

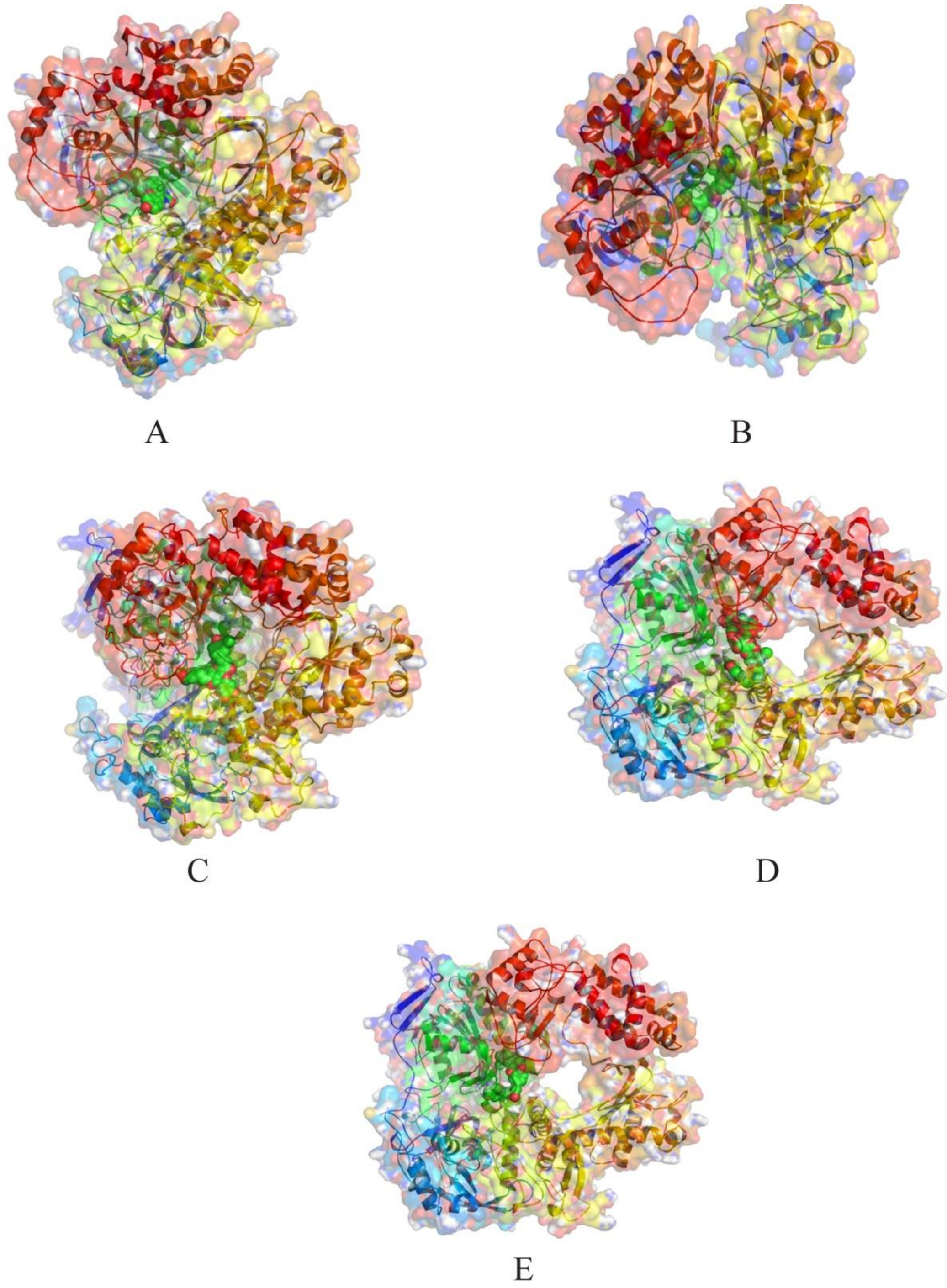

Figure. S2. 3D complex of the drug target with (A) Disalicyloyl curcumin, (B) Ferrocenyl curcumin (C) Curcumin dimer 1 (D) Curcumin dimer 2 (E)N-(4 Methoxyphenylpyrazole) Curcumin.
